# Mobile Phone–Based Behavioral Interventions in Pregnancy to Promote Maternal and Fetal Health in High-Income Countries: Systematic Review

**DOI:** 10.2196/15111

**Published:** 2020-05-28

**Authors:** Tasmeen Hussain, Patricia Smith, Lynn M Yee

**Affiliations:** 1 Department of Internal Medicine Northwestern University Feinberg School of Medicine Chicago, IL United States; 2 Division of Maternal Fetal Medicine Department of Obstetrics and Gynecology Northwestern University Feinberg School of Medicine Chicago, IL United States

**Keywords:** mHealth, mobile health, pregnancy, smartphone, text messaging, mobile applications, software, chronic disease, health behavior

## Abstract

**Background:**

Chronic diseases have recently had an increasing effect on maternal-fetal health, especially in high-income countries. However, there remains a lack of discussion regarding health management with technological approaches, including mobile health (mHealth) interventions.

**Objective:**

This study aimed to systematically evaluate mHealth interventions used in pregnancy in high-income countries and their effects on maternal health behaviors and maternal-fetal health outcomes.

**Methods:**

This systematic review identified studies published between January 1, 2000, and November 30, 2018, in MEDLINE via PubMed, Cochrane Library, EMBASE, CINAHL, PsycINFO, Web of Science, and gray literature. Studies were eligible for inclusion if they included only pregnant women in high-income countries and evaluated stand-alone mobile phone interventions intended to promote healthy maternal beliefs, behaviors, and/or maternal-fetal health outcomes. Two researchers independently reviewed and categorized aspects of full-text articles, including source, study design, intervention and control, duration, participant age, attrition rate, main outcomes, and risk of bias. Preferred Reporting Items for Systematic Reviews and Meta-Analyses guidelines were followed, and the study was registered in PROSPERO before initiation.

**Results:**

Of the 2225 records examined, 28 studies were included and categorized into 4 themes: (1) gestational weight gain, obesity and physical activity (n=9); (2) smoking cessation (n=9); (3) influenza vaccination (n=2); and (4) general prenatal health, preventive strategies, and miscellaneous topics (n=8). Reported sample sizes ranged from 16 to 5243 with a median of 91. Most studies were performed in the United States (18/28, 64%) and were randomized controlled trials (21/28, 75%). All participants in the included studies were pregnant at the time of study initiation. Overall, 14% (4/28) of studies showed association between intervention use and improved health outcomes; all 4 studies focused on healthy gestational weight. Among those, 3 studies showed intervention use was associated with less overall gestational weight gain. These 3 studies involved interventions with text messaging or an app in combination with another communication strategy (Facebook or email). Regarding smoking cessation, influenza vaccination, and miscellaneous topics, there was some evidence of positive effects on health behaviors and beliefs, but very limited correlation with improved health outcomes. Data and interventions were heterogeneous, precluding a meta-analysis.

**Conclusions:**

In high-income countries, utilization of mobile phone–based health behavior interventions in pregnancy demonstrates some correlation with positive beliefs, behaviors, and health outcomes. More effective interventions are multimodal in terms of features and tend to focus on healthy gestational weight gain.

## Introduction

### Background

Pregnancy and the postpartum period are times of rapid medical, social, and behavioral changes for women and their families. This period is perceived to be a *window of opportunity* for health interventions because many women have enhanced access to health care during pregnancy and may have increased motivation to improve their health during this time. Healthy maternal behaviors have been shown to improve the risk of pregnancy-related morbidities [1]. For example, smoking cessation, exercise, and healthy weight gain in pregnancy have all been linked to better maternal and fetal health [2-4].

Chronic disease is a particularly important arena. Per the Centers for Disease Control and Prevention, although the rate of maternal death related to traditional risk factors such as hemorrhage, hypertensive disorders of pregnancy, and anesthesia complications in the United States is decreasing, mortality related to cardiovascular disease (CVD), cerebrovascular accidents, and other medical conditions continues to increase [5]. Cardiovascular conditions were responsible for more than one-third of all pregnancy-related deaths in the United States between 2011 and 2016. Thus, pregnancy is an important time to improve health behaviors, such as promoting healthy gestational weight gain and managing chronic disease. However, changes to health behaviors often require intensive provider support, consistent follow-up, and frequent counseling that are difficult to maintain during short outpatient visits. These requirements may be supported by technology.

Many health behavior and lifestyle interventions have incorporated technology in various areas of chronic disease management [6,7]. In particular, the field of mobile health (mHealth) has recently seen rapid growth. mHealth refers to the use of mobile technologies including mobile phones, personal digital assistants, and even tablet computers to improve patient health. Outside of pregnancy, a growing amount of literature suggests that mHealth and other digital interventions are feasible, acceptable, and may promote improved health behaviors [8-10]. An estimated 76% of people in high-income countries own a mobile phone, and 87% use the internet [11,12]. Furthermore, a more focused study of pregnant women in the United States showed that 88% had access to a mobile phone, and 89% had access to the internet [13]. These data suggest both are promising media for use with pregnant women in the management of chronic conditions in high-income environments.

Past studies suggest women are interested in receiving health information on the Web and are comfortable with using their mobile phones [14]. However, research on the use of mHealth in pregnancy has been broad and heterogeneous. Much of the research done is with small groups in low- or middle-income countries or utilizes a *telemedicine* format, defined as technology-facilitated direct communication with medical professionals [15-22]. There remains a lack of organized discussion on mHealth interventions in pregnancy that are tailored or self-maintaining as well as on studies of women in high-income countries, where access to mobile phones is the greatest and women are highly affected by chronic disease

### Objective

The objective of this study was to systematically evaluate mHealth interventions used in pregnancy in high-income countries and their effects on maternal health behaviors and maternal-fetal health outcomes.

## Methods

### Study Registration

Before performance of this search, information about the study proposal was published electronically in the University of York PROSPERO register of systematic reviews [23]. The authors followed all guidelines for the Preferred Reporting Items for Systematic Reviews and Meta-Analyses (PRISMA) statement [24].

### Eligibility Criteria, Information Sources, and Search Strategy

We conducted a systematic review of studies on mobile phone–based mHealth interventions designed for pregnant women. A research librarian (PS) was primarily responsible for a comprehensive literature search. We included English-language articles with a patient population that included pregnant women who utilized pregnancy-related mobile phone interventions during their pregnancy. In addition, we limited our studies to those performed in developed or high-income countries as defined by The World Economic Situation and Prospects 2012 of the United Nations [25]. Study types included meta-analyses, systematic reviews, randomized controlled trials (RCTs) including randomized crossover trials and cluster randomized trials, and nonexperimental observational studies. The assessed interventions were stand-alone mobile phone–based interventions including, but not limited to, mobile phone apps, text messaging, games, and information services. We excluded studies that used technology interventions aimed solely at communication between patients and clinicians without a stand-alone educational, motivational, or interactive component (such as telemedicine portals or electronic medical record–based portals for use with mobile phones), and interventions that were not primarily intended for mobile phone use, eg, websites. Studies were excluded if they focused solely on neonatal health, such as neonatal feeding support interventions or growth tracking tools. Studies were also excluded if they were exclusively published as abstracts or conference proceedings without a full peer-reviewed manuscript. Finally, studies were excluded if they were solely meant to evaluate feasibility or desirability of hypothetical interventions or supplied outcomes with fewer than 2 weeks of intervention use.

We searched MEDLINE via PubMed, EMBASE, Web of Science, Cochrane Database of Controlled Trials, CINAHL, and PsycINFO databases from January 1, 2000 to November 30, 2018. We began with the MEDLINE search and translated to the appropriate syntax for each of the other databases, using controlled vocabulary when possible. Search terms related to pregnancy, mobile interventions, and select behaviors (including smoking cessation, weight loss, and diabetes management) were included. Full search strategies can be found in Multimedia Appendix 1, and a completed PRISMA checklist is found in Multimedia Appendix 2.

### Study Selection

Titles and abstracts of studies were read by 2 independent reviewers (TH and LY) on two online abstract organizers (abstrackr: [26] and Rayyan [27]). Discordant assessments were resolved by discussion between reviewers or with the involvement of a third author (PS) when necessary.

Studies were then divided into 4 subgroups based on their primary clinical focus: (1) gestational weight gain, obesity and physical activity; (2) smoking cessation; (3) influenza vaccination; and (4) general prenatal health, preventive strategies, and miscellaneous topics. For all study types, data extraction was standardized to include source, study design, number of participants in the intervention and control groups, intervention and control descriptions, duration, participant age and other details if available, attrition rates, and main outcomes.

### Data Extraction

Two authors (TH and LY) simultaneously reviewed all abstracts for inclusion using Abstrackr and Rayyan, as described above. EndNote X7.2 (EndNote, Clarivate Analytics, Philadelphia, Pennsylvania, United States) was used to identify and remove duplicate records. Two searches were conducted; the initial search reviewed literature to 2016 and an updated search reviewed more recent literature until November 30, 2018. Once relevant abstracts were agreed upon, full-text analysis of included abstracts was then performed by the same authors. In addition, review of the bibliographies of included full-text articles were reviewed for additional eligible articles. Relevant articles meeting the final inclusion criteria were then abstracted in-depth for bias, study quality, and overall findings.

### Assessment of Risk of Bias

Bias was evaluated by 2 independent reviewers (TH and LY). We applied specific tools for assessment of risk of bias tailored to each study type. For observational studies (not randomized controls), we used 1 of 2 National Institutes of Health (NIH) Quality Assessment Tools (NIH QAT), which consisted of 12 items to assist raters in formulating a holistic final quality assessment [28]. If a study had a control but was not randomized, the Quality Assessment of Controlled Intervention Studies was used; if no control was available, the Quality Assessment Tool for Before-After (Pre-Post) Studies with No Control Group was used. For RCTs, we used the Cochrane Risk of Bias tool [29]. Studies were rated independently by 2 reviewers (TH and LY). Disagreements were resolved by discussion between reviewers or with the involvement of a third author (PS) when necessary.

### Data Synthesis

Data were collected to be primarily presented descriptively. We considered a meta-analysis or pooling of data if sufficient homogeneity in measured outcomes were to be observed, but the evaluation of data demonstrated heterogeneity that precluded such analyses.

## Results

### Study Selection

**Figure 1:**
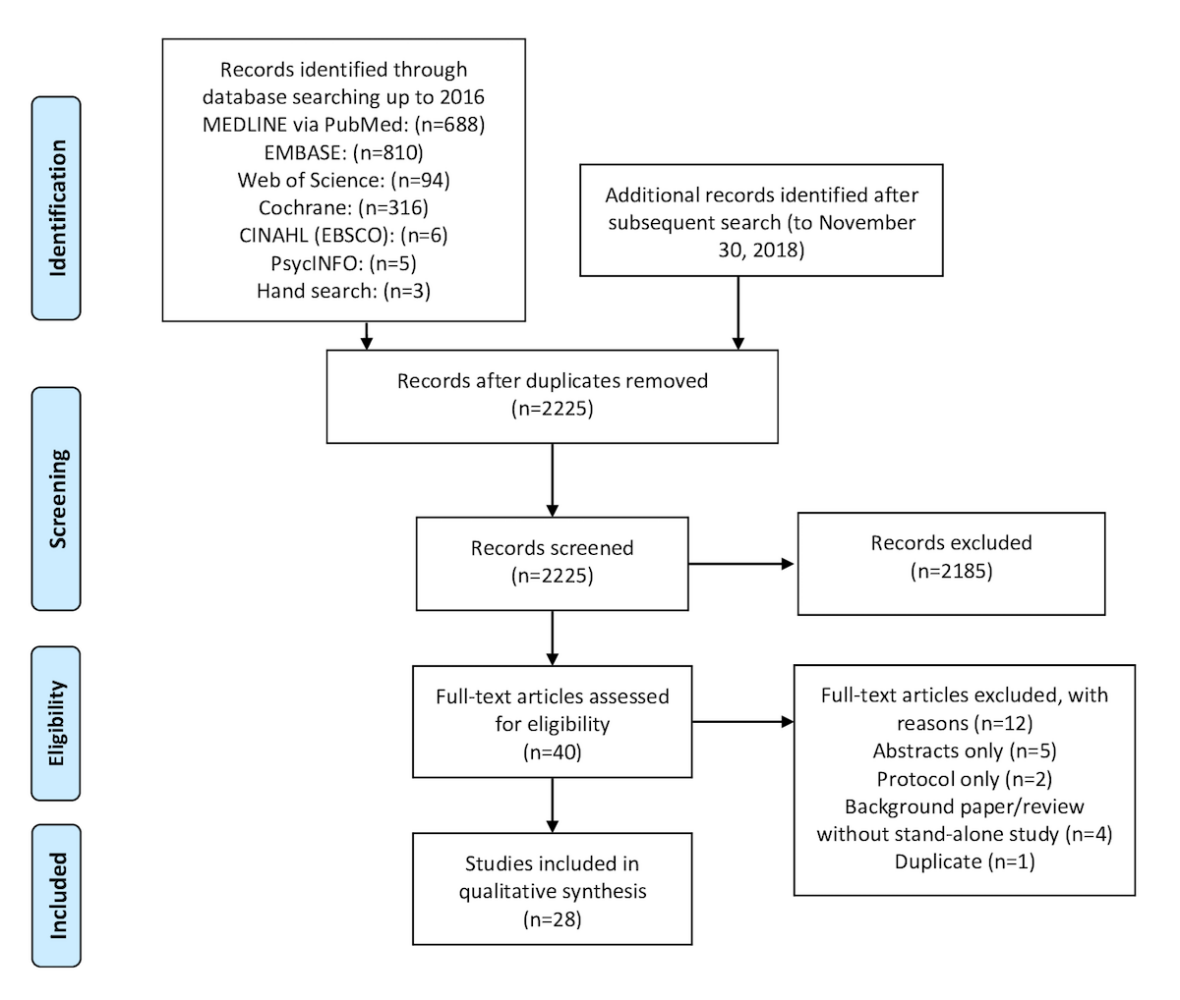
Preferred Reporting Items for Systematic Reviews and Meta-Analyses flow sheet.

An electronic search as described previously revealed a total of 2225 titles and abstracts after the removal of duplicates. After full-text evaluation, a total of 28 studies met the criteria for inclusion. An adapted PRISMA study flowchart is shown in Figure 1.

### Study Characteristics and Synthesis of Results

Included studies fell into 4 categories: (1) gestational weight gain, obesity, and physical activity (n=9); (2) smoking cessation (n=9); (3) influenza vaccination (n=2); and (4) general prenatal health, preventive strategies, and miscellaneous topics (n=8). Reported sample sizes ranged from 16 to 5243 with a median of 91. All participants in the included studies were pregnant at the time of study initiation.

Tables 1 and 2 outline studies focused on gestational weight gain, obesity, and physical activity [28,30-37]. Of the 9 eligible studies, 2 used exclusively text messages, 4 utilized text messages in conjunction with other technology, and 3 utilized mobile phone apps without text messages. One included study was not randomized, whereas the remainder were RCTs. Outcomes varied widely among studies. Two studies showed that intervention participants were significantly less likely to exceed healthy gestational weight gain during pregnancy (37% vs 66%; *P*=.03) [31]^,^ and (58% vs 85%; *P*=.04) [36], but one found no such difference [33]. Three interventions were associated with less overall gestational weight gain in intervention users over the study period [31,33,37]. Notably, each of these interventions were multimodal and incorporated at least one additional communicative technology (Facebook or emails) alongside its main intervention (text messages or an app). The 2 studies that evaluated gestational weight gain and utilized interactive text messages or an app alone exhibited no difference in gestational weight gain compared with controls [34,36]. Studies also differed regarding behavior change. Although some showed improvements in behavior analogs such as increased self-reported exercise [37] and less reduction in physical activity during pregnancy compared with prepregnancy [33], others showed no such relationship [32,34,35]. There was no difference in incidence of gestational diabetes in any study [31,37]. In terms of cost, 1 study did find that a mobile app compared with a parallel intervention requiring in-person counseling by health coaches was significantly less expensive (US $97 vs US $347) [36]. In this study, both remote and in-person interventions were associated with lower proportion of excess gestational weight gain when compared with controls.

**Table 1:** Design of trials with a focus on gestational weight gain, obesity, and physical activity.

Reference	Setting/country/population	Study design	Experimental arm vs control arm(s), n	Intervention description and control	Duration
Soltani et al (2015) [28]	Prenatal clinic/Doncaster, England/BMI>30; 8-10 weeks’ gestation	Observational	Intervention vs control: 16 vs 15	MOMTech text messages: 14 motivational text messages per week, food and activity diary, goal setting, and consultation visits vs usual care	Until 6 weeks postpartum
Choi et al (2016) [30]	Prenatal clinics and community/San Francisco, CA, United States/Sedentary; 10-20 weeks’ gestation	RCT^a^	Intervention vs control: 15 vs 15	Mobile app: Fitbit-enhanced daily message as text message or short video script, activity diary, and automated feedback vs Fitbit only	12 weeks
Herring et al (2016) [31]	Prenatal clinics/Philadelphia, Pennsylvania, United States/African American; <20 weeks’ gestation; BMI 25-45	RCT	Intervention vs control: 33 vs 33	Behavior change goals, interactive self-monitoring text messages, biweekly health coach calls, and skills training and support through Facebook vs usual care	Until 36 weeks’ gestation
Dodd (2017) [32]	Public maternity hospitals/Adelaide, South Australia/10-20 weeks’ gestation	RCT	Intervention vs control: 77 vs 85	Interactive mobile phone app with information about dietary guidelines and physical activity guidelines during pregnancy; also encouraged women to set dietary and physical activity goals and monitor their progress vs lifestyle advice only	Until 36 weeks’ gestation
Willcox (2017) [33]	Academic maternity hospital/Melbourne, Australia/10-17 weeks’ gestation; prepregnancy BMI>25	RCT	Intervention vs control: 45 vs 46	txt4two: Tailored text messages, Web-based app, video messages, and Facebook chat room and brochure vs brochure only	Until 36 weeks’ gestation
Pollak (2014) [34]	Prenatal clinics/Durham, NC, United States/prepregnancy BMI=25-40; 12-21 weeks’ gestation	RCT	Intervention vs text4baby: 22 vs 11	Preg CHAT texts: interactive 3 times weekly texts regarding behaviors–step counts, sweetened drinks, fruits/vegetables, and eliminating fast foods vs text4baby alone	16 weeks
Huberty (2017) [35]	Online/US residents/8-16 weeks’ gestation; low physical activity	RCT	3 intervention groups: Plus One group (21); Plus Six (20); Plus Six Choice (18); and standard group (21)	3 intervention groups with variations on general and physical activity texts received per week: Plus One, Plus Six, Plus Six Choice; participants also received Fitbit flex to track sleep and exercise data. All were compared with the *standard* group, which was three text4baby SMS per week at noon	Until 40 weeks’ gestation
Redman (2017) [36]	Various clinics/United States/BMI=25-39.9; first trimester of pregnancy	RCT	2 intervention groups: In person (18), Remote (19), and control (17)	2 intervention groups: In person—dietary intake advice, exercise advice, paper weight graph and counseling provided by health coaches; Remote—same information as above provided in a mobile app format with electronic data capture; both compared with usual care from obstetrician	Until delivery
Kennelly (2018) [37]	Maternity hospital/Dublin, Ireland/BMI=25-39.9; 10-15 weeks’ gestation	RCT	Intervention vs usual care: 278 vs 287	A mobile phone app with low glycemic index recipes, an exercise advice section, and a home page with tips and encouraging thought of the day. Also received emails every 2 weeks and two face-to-face hospital visits vs usual care	Until 34 weeks’ gestation

^a^RCT: randomized controlled trial.

**Table 2:** Outcomes and bias of trials with a focus on gestational weight gain, obesity, and physical activity.

Reference	Participant age (years), mean (SD)	Attrition rate	Main outcomes	Bias tool	Bias rating	Bias reasoning
Soltani et al (2015) [28]	29.1 (5.4) for IG^a^ vs 31.7 (5.8) for CG^b^	13% (2/16)	No significant difference in mean GWG^c^ (5.6 vs 9.7 kg) No significant difference in percentage of participants who exceeded the IOM^d^ upper limit of GWG for obese women (28% vs 50%)	NIH QAT^e^	Fair risk	Small sample size
Choi et al (2016) [30]	32.9 (2.5) for IG vs 34.5 (2.5) in CG	40% to daily messages, 33% to activity diary	Significantly less “Lack of energy as a barrier to being active,” at week 12 in IG (*P*=.02) No difference between groups in change in weekly mean steps (*P*=.23) No change in numerous outcomes including CES-D^f^ score, severity of pregnancy symptoms, self-efficacy	Cochrane ROBT^g^	Low risk	N/A^h^
Herring et al (2016) [31]	25.9 (4.9) for IG vs 25.0 (5.7) for CG	Unclear	Significantly greater percentage of IG kept within IOM guidelines for GWG (37% vs 66%; *P*=.03) Significant adjusted mean difference in total GWG in IG, early pregnancy to delivery (8.7 vs 12.3 kg; *P*=.046) No significant difference in mean birth weight or babies small or large for gestational age. No difference in percentage of women with GDM^i^	Cochrane ROBT	Low risk	N/A
Dodd (2017) [32]	30.87 (5.07) for IG vs 31.01 (6.16) for CG	38.2% (62/162)	No significant difference in self-reported Healthy Eating Index scores, macronutrient and food group intake, or physical activity	Cochrane ROBT	High risk	High attrition, self-report, and women knew allocations
Willcox (2017) [33]	33.0 (3.4) for IG vs 32.0 (5.1) for CG	9.0% (9/100)	Significantly less GWG with txt4two (7.8 vs 9.7 kg; adjusted *P*=.04) Significantly fewer txt4two women reduced their minutes of total daily physical activity over the course of the intervention (*P*=.001) No significant difference in proportion of women exceeding IOM GWG guidelines. (47% vs 61%; adjusted *P*=.07) No significant differences in self-reported consumption of food groups	Cochrane ROBT	High risk	Women not blinded, self-reported exercise
Pollak (2014) [34]	29 (5) for IG vs 32 (2) in CG	30% (10/33)	No significant difference in mean weight gain, physical activity level outcomes, or nutrition score	Cochrane ROBT	High risk	High proportional attrition, low sample size. Possibly randomized by study staff
Huberty (2017) [35]	31.05 (5.52) for Plus One vs 31.48 (5.44) for Plus Six vs 31.44 (4.16) for Plus Six Choice vs 30.83 (5.22) for standard	14% (13/93)	All 3 IGs were consolidated; when compared with controls, no difference in linear trajectories or quadratic trajectories regarding active time, light intensity time, and steps	Cochrane ROBT	Fair risk	Not blinded
Redman (2017) [36]	29.0 (4.2) for remote vs 29.2 (4.8) for in person vs 29.5 (5.1) for CG	Unclear	Significantly lower proportion of women with excess GWG in the remote group compared with usual care groups (58% vs 85%; *P*=.04) No significant difference in GWG between the remote group and usual care (least squares mean 10.0 vs 12.8 kg; *P*=.07) Significantly less intervention cost for remote compared with in-person group (US $97 vs US $347; *P*<.001)	Cochrane ROBT	High risk	Randomized by unblinded intervention staff
Kennelly (2018) [37]	32.8 (4.6) for IG vs 32.1 (4.2) for CG	11.9% (67/565)	No significant difference in incidence of GDM (15.4% vs 14.1%; *P*=.71) Significantly less GWG in IG (8.9 vs 10 kg; *P*=.02) Significantly lower dietary glycemic load (*P*=.02) and increased exercise in IG (*P*=.02) after multiple correction testing	Cochrane ROBT	Fair risk	Self-reported exercise and food outcomes; neither participants nor researchers blinded

^a^IG: intervention group.

^b^CG: control group.

^c^GWG: gestational weight gain.

^d^IOM: Institute of Medicine.

^e^NIH QAT: National Institutes of Health Quality Assessment Tool.

^f^CES-D: Center for Epidemiologic Studies Depression Scale.

^g^N/A: not applicable.

^h^ROBT: risk of bias tool.

^i^GDM: gestational diabetes mellitus.

Tables 3 and 4 outline interventions to address smoking cessation during pregnancy [38-46]. Of the 9 studies, 7 used exclusively text messages, and the remaining 2 studies used mobile phone apps. Overall, outcomes were sparse regarding the ability of interventions to affect smoking cessation. Although 2 small uncontrolled studies showed a decrease in cigarettes smoked over the course of intervention [38] and more than 70% achievement of nonsmoking by the end of the intervention [39], the studies that employed control arms showed no difference in outcomes. These outcomes varied but included self-reported abstinence, biochemically reported abstinence, and number of smoke-free days [40-46]. In 1 study, using text messages as the intervention mode was associated with increased self-efficacy, determination to quit smoking in pregnancy, and setting a quit date [42].

Tables 5 and 6 highlight the 2 studies of interventions to improve influenza vaccination rates [47,48]. Both utilized text messages alone. There was no difference in influenza vaccination rates in intervention vs control groups in either study.

Tables 7 and 8 outline the remaining 8 studies, which focused on general prenatal health, preventive strategies, and miscellaneous topics [49-56]. Four studies employed text messages alone, and 4 used mobile phone apps. In this sphere, interventions were most associated with improvements in health beliefs [49] and behaviors including self-reported attempts to eat more nutritious food [50], belief that taking prenatal vitamins will improve the health of the fetus [54], and belief that the participant is prepared to be a new mother [54]. There was also a significant association between intervention use and attending a prenatal visit at least 6 months before delivery in 1 controlled study [51]. In 1 study without formal controls, there was a higher rate of clinic attendance in intervention users (84%) compared with that for the general clinic population (50%) [52]. In this study, attendance was even higher (89%) than in those who scheduled transportation through a free rideshare service facilitated through the app. Among these studies, there was no difference in any measured health outcomes including cesarean delivery and neonatal intensive care unit admission [51], hypertensive disorders of pregnancy, gestational weight gain, delivery outcomes [53], and beliefs and behaviors around smoking and alcohol [54,55]. One unique study employed a mobile phone app to improve rates of perineal massage in Japan; this intervention was not associated with any difference in rates of practice of perineal massage, perineal lacerations, or episiotomy rates [56].

**Table 3:** Design of trials with a focus on smoking cessation.

Reference	Setting/country/population	Study design	Experimental arm vs control arm(s), n	Intervention description and control	Duration
Abroms et al (2015) [38]	Online/United States/current smoker or recently quit (<4 weeks ago), <30 weeks’ gestation	Observational	Intervention (20), no control arm	Quit4baby text messages: 1-5 messages per day in reference to chosen quit date; also included interactive keyword-based support messages. Participants continued to receive text4baby messages concurrently	4 weeks
Fujioka et al (2012) [39]	Obstetrics consultations/Yamaguchi prefecture, Japan/current smokers, >20 weeks’ gestation	Observational	Intervention (52), no control arm	Mobile phone e-learning program: smoking cessation education, ability to set quit date, ability to select who will help quit smoking, record of declaration of quitting smoking	3 months
Abroms (2017) [41]	Prenatal clinics/Washington DC, United States/current smoker or recently quit (<2 weeks ago)	RCT^a^	Intervention (55) vs control (44)	SmokefreeMOM: Tailored and interactive texts 3-6 times per day regarding smoking including setting a quit date, self-efficacy, and expectations regarding quitting vs usual care	3 months
Naughton et al (2012) [42]	Prenatal clinics/England/current smokers, <21 weeks’ gestation	RCT	Intervention (102) vs control (105)	MiQuit text messages: Tailored text messages 0-2 times/day at random intervals as well as *instant-response* supportive texts for help or lapses in behavior and tailored leaflet vs untailored leaflet	3 months
Pollak et al (2013) [43]	Prenatal clinics/United States/current smokers, 10-30 weeks’ gestation	RCT	Intervention (16) vs control (15)	Scheduled gradual reduction SMS: Gradual program to reduce smoking to 0 cigarettes by the 4th week. Support messages included up to 5 messages per day about various smoking cessation topics as well as setting a quit date vs support messages alone	5 weeks
Tombor (2018) [44]	Online/England/current smokers	RCT	565 randomized to one of 32 groups in full factorial design, randomized to *full* or *minimal* version of each module	SmokeFree Baby App assessed 5 modules: identity, health information, stress management, face-to-face support, behavioral substitution	4 weeks
Abroms (2017) [40]	Online/United States; current smokers	RCT	Intervention (250) vs control (247)	Quit4baby: Tailored and interactive texts 1-8 times/day regarding smoking including setting a quit date, self-efficacy, and expectations regarding quitting. Was employed in addition to Text4baby. Compared with Text4baby alone	3 months
Forinash (2018) [45]	Prenatal clinic, St. Louis, MO, United States/current smokers	RCT	Intervention (14) vs control (16)	Text messages every several days in a tapering pattern with encouragement to stop smoking vs usual care	8 weeks
Naughton (2017) [46]	Prenatal clinics, England/<25 weeks’ gestation; current smokers	RCT	Intervention (203) vs control (204)	MiQuit: an automated 12-week advice and support program for quitting smoking delivered by SMS text message. Tailored to desired themes including gestation, motivation to quit, self-efficacy, and partner’s smoking status vs usual care	Until 36 weeks’ gestation

^a^RCT: randomized controlled trial.

**Table 4:** Outcomes and bias of trials with a focus on smoking cessation.

Reference	Participant age (years), mean (SD)	Attrition rate	Main outcomes	Bias tool	Bias rating	Bias reasoning
Abroms et al (2015) [38]	28.1 (6.1) for total sample	35% (7/20)	Cigarettes smoked decreased from 7.6 (4.9) to 2.4 (1.8) after 4 weeks but was not significant	NIH QAT^a^	Fair risk	No pre- to postanalysis, multiple measurements not taken, high loss to follow-up, high attrition
Fujioka et al (2012) [39]	25.9 (4.7) for total sample	7.7% (4/52)	71.1% of participants achieved nonsmoking Confidence to continue not smoking increased in both groups (those who ended up smoking, and those who quit smoking)	NIH QAT	Fair risk	Not all eligible participants were enrolled, measurements not taken multiple times
Abroms (2017) [41]	27.18 (4.98) for IG^b^ vs 28.25 (4.78) for CG^c^	26% (26/99)	No significant differences in any smoking-related outcomes including biochemically confirmed 7-day PPA^d^, self-reported 7-day and 30-day abstinence, consecutive days quit, quit attempts, and changes in cigarettes smoked/day	Cochrane ROBT^e^	High risk	No information about blinding; randomization scheme changed in the middle of study
Naughton et al (2012) [42]	27.2 (6.4) for IG vs 26.5 (6.2) for CG	11% (23/207)	Significantly higher overall self-efficacy, habitual self-efficacy, social self-efficacy and determination to quit smoking in pregnancy in IG Significantly higher probability to set a quit date in intervention group (45% vs 30%) No difference in outcomes including self-reported point prevalence at 3, 7, and 12 weeks, or making at least one 24 hour quit attempt	Cochrane ROBT	Low risk	N/A^f^
Pollak et al (2013) [43]	29 (6) for IG and 27 (6) for CG	6% (2/31)	No change in 7-day point prevalence (7.5% vs 13.4%) or cigarettes smoked	Cochrane ROBT	High risk	Blinding and randomization strategies unclear
Tombor (2018) [44]	27.3 (5.5) for total sample	68.9% (389/565)	No module was associated with fewer smoke-free days	Cochrane ROBT	High risk	Very high attrition rate; of note, all lost to follow-up were assumed to be smokers
Abroms (2017) [40]	26.68 (5.94) for IG vs 25.95 (5.74) for CG	28.2% (140/497)	Overall, no significant difference in biochemically confirmed 7-day PPA at 3-month follow-up (39% vs 27%) IG vs CG	Cochrane ROBT	Fair risk	Self-reporting patients knew which group they were in
Forinash (2018) [45]	Not provided	39% (19/49)	No significant difference was found in eCO^g^-verified cessation (57.1% vs 31.3%; *P*=.15), eCO below 8 ppm at ≥1 visit (64.3% vs 37.5%; *P*=.14), or in birth outcomes	Cochrane ROBT	High risk	High attrition
Naughton (2017) [46]	26.6 (5.7) for IG vs 6.4 (5.7) for CG	35.9% (146/407)	No difference in self-reported, later biochemically confirmed, abstinence in late pregnancy	Cochrane ROBT	Fair risk	Moderately high attrition; of note, those lost to follow-up assumed to be smokers

^a^NIH QAT: NIH Quality Assessment Tool.

^b^IG: intervention group.

^c^CG: control group.

^d^PPA: point prevalence abstinence.

^e^ROBT: risk of bias tool.

^f^N/A: Not applicable.

^g^eCO: exhaled carbon monoxide.

**Table 5:** Design of trials with a focus on influenza vaccination.

Reference	Setting/country/population	Study design	Experimental arm vs control arm(s), n	Intervention description and control	Duration
Moniz et al (2013) [47]	Prenatal clinic/Pittsburgh, Pennsylvania, United States/<28 weeks’ gestation	RCT^a^	Intervention (104) vs control (100)	12 weekly text messages with information about the benefits and safety of influenza vaccine in pregnancy vs control messages about general pregnancy health alone	12 weeks, up to 6 weeks postpartum
Yudin (2017) [48]	Prenatal clinic/Toronto, Canada/any gestational age	RCT	Intervention (153) vs control (164)	Text messages twice weekly × 4 weeks emphasizing pregnant women’s susceptibility to influenza, effectiveness of vaccine, safety of vaccines, and that vaccines are recommended for pregnant women vs usual care	Until 6 weeks postpartum

^a^RCT: randomized controlled trial.

**Table 6:** Outcomes and bias of trials with a focus on influenza vaccination.

Reference	Participant age (years), mean (SD)	Attrition rate	Main outcomes	Bias tool	Bias rating	Bias reasoning
Moniz et al (2013) [47]	Ranged 13-49	23% (46/204)	No difference in influenza vaccination rate (33% vs 31%)	Cochrane ROBT^a^	Low risk	N/A^b^
Yudin (2017) [48]	32.2 (4.5) for IG^c^ vs 32.4 (4.9) for CG^d^	10.7% (34/317)	No difference in influenza vaccination rate (31% vs 27%; *P*=.51)	Cochrane ROBT	Low risk	N/A

^a^ROBT: risk of bias tool.

^b^N/A: not applicable.

^c^IG: intervention group.

^d^CG: control group.

**Table 7:** Design of trials with a focus on general health, preventive health, health beliefs, and other topics.

Reference	Topic	Setting/country/population	Study design	Experimental arm vs control arm(s), n	Intervention description and control	Duration
Moniz et al (2015) [49]	Preventive health behaviors (smoking cessation, condom use, nutrition optimization, seat belt use, breastfeeding)	Prenatal clinic/Pittsburgh, Pennsylvania, United States/<28 weeks’ gestation	Observational	Intervention (171), no control arm	General preventive health text messages regarding tobacco cessation, sexually transmitted disease prevention, daily vitamin use, seat belt use, dietary discretion and breastfeeding	12 weeks
Dalrymple et al (2013) [50]	General prenatal health topics	Prenatal clinic/Philadelphia, Pennsylvania, United States/no special population	Observational	Intervention (31), no control arm	Twice weekly text messages delivered alongside text4baby messages on days text4baby messages were not sent	Unclear
Bush (2017) [51]	Numerous including weight, milestones, Wyoming-specific resources	Online/Wyoming state, United States/Medicaid users	Observation	Intervention (85) vs non–app-Medicaid members (5158)	Wyhealth Due Date Plus: mobile phone app that includes information on 70 health risk factors, provides pregnancy timeline, weight tracker, and appointment reminders	6 months
Krishnamurti (2017) [52]	Numerous, including nutrition, routine prenatal care, violence, smoking, preterm labor	Prenatal clinic/Pittsburgh, Pennsylvania, United States/Medicaid-qualifying women	Observation	Intervention (16), no control arm	My Healthy Pregnancy App: Interactive application that gathered data regarding risk factors and delivered patient-specific risk feedback and recommendations. Could also arrange for Uber rides to clinic	3 months or until delivery
Ledford (2016) [53]	General obstetric care, health literacy	Prenatal clinic/Bethesda, MD, United States/10-12 weeks’ gestation	RCT^a^	Intervention (87) vs control (86)	Mobile app for journaling with space for recording weight, blood pressure, and experience between prenatal appointments vs spiral notebook alone	Until 32 weeks’ gestation
Evans et al (2014) [54]	General prenatal health topics	Army Medical Center/Tacoma, WA, United States/<14 weeks’ gestation	RCT	Intervention (498) vs control (498)	Text messages: 3 automated, tailored text messages per week vs usual care	4 weeks
Evans et al (2012) [55]	General prenatal health topics	Prenatal clinic/Fairfax county, VA, United States/largely low-income	RCT	Intervention (48) vs control (38)	Automated, tailored text messages vs usual care	28 weeks’ gestation
Takeuchi (2016) [56]	Perineal massage	Prenatal clinic/Tokyo, Japan/30-33 weeks’ gestation	RCT	Intervention (81) vs control (80)	Mobile phone website underlining effects of perineal massage, massage technique, support through peer group, communication with professional, and reminders/encouragement vs leaflet alone	Until delivery

^a^RCT: randomized controlled trial.

**Table 8:** Outcomes and bias of trials with a focus on general health, preventive health, health beliefs, and other topics.

Reference	Participant age (years), mean (SD)	Attrition rate	Main outcomes	Bias tool	Bias rating	Bias reasoning
Moniz et al (2015) [49]	24.0 (4.5)	8% (13/171)	Participants agreed that receiving text messages changed their beliefs about targeted preventive health behaviors: Smoking (50%) Sexually transmitted disease prevention (72%) Prenatal vitamins (83%) Seat belt use (68%) Nutritious food intake (84%) Breastfeeding (68%)	NIH QAT^a^	Fair risk	No before/after or multiple measurements taken
Dalrymple et al (2013) [50]	Unclear	84% (26/31) for posttest; 35% (11/31) for any monthly form	100% agreed “I tried to eat better for myself and the baby.” 60% agreed “I understood what was happening to my body better.”	NIH QAT	High risk	No before/after or multiple measurements taken, small sample size, high attrition
Bush (2017) [51]	Unclear	Unclear	Significant association between app use and completion of a prenatal visit at least 6 months before delivery (OR^b^ 1.76; *P*=.02) Borderline significant association between app use and low birth weight (OR 0.25; *P*=.06) No association between app use and cesarean delivery or NICU^c^ admission	NIH QAT	High risk	Used a comparison that was not randomly selected (self-selected app users)
Krishnamurti (2017) [52]	Median 24, range (18-35)	0% (0/16)	Intervention users reported higher intention to breastfeed at 2 months (t_13_=−4.16; *P*=.001) and 3 months (t_15_=−2.76; *P*=.01) No statistical significance in intention to use prenatal vitamins Clinic attendance rate was higher in participants than nonparticipant clinic patients (84% vs 50%) Attendance was even higher (89%) among those who scheduled free Uber transportation	NIH QAT	High risk	Sample size too low
Ledford (2016) [53]	29.29 (4.8) for IG^d^ vs 29.37 (4.83) for CG^e^	27% (46/173)	Mobile group reported more frequent use (*P*=.04) and greater patient activation (*P*=.02) than the notebook group No difference in biometrics including blood pressure control, weight gain, delivery outcomes	Cochrane ROBT^f^	Fair risk	Unclear how randomization occurred, patients not blinded
Evans et al (2014) [54]	26.53 (SD not noted)	51.3% (484/943)	Significantly more of the intervention group agreed that “Taking prenatal vitamins will improve the health of my developing baby” (OR 1.91; *P*=.02) No difference in outcomes including self-reported smoking, consumption of alcoholic beverages or fruit and vegetable consumption	Cochrane ROBT	Fair risk	Selective reporting, high attrition
Evans et al (2012) [55]	27.6 (SD not noted)	27% (33/123)	Significantly more of the intervention group agreed that “I am prepared to be a new mother” (OR 2.73; *P*=.04) No difference in outcomes including beliefs that smoking will harm the developing baby, that drinking alcohol will harm the developing baby, and that taking prenatal vitamins will improve the health of the developing baby	Cochrane ROBT	Fair risk	Unclear blinding of participants and personnel; incomplete outcome data
Takeuchi (2016) [56]	32.7 (4.59) for IG vs 32.5 (4.18) for CG	40% (65/161)	No difference in practice of perineal massage, perineal lacerations, or episiotomy rates	Cochrane ROBT	High risk	High attrition rate, self-assessment by unblinded participants, unclear randomization

^a^NIH QAT: NIH Quality Assessment Tool.

^b^OR: odds ratio.

^c^NICU: neonatal intensive care unit.

^d^IG: intervention group.

^e^CG: control group.

^f^ROBT: risk of bias tool.

### Risk of Bias in Included Studies

Bias ratings for all studies are included above in Tables 1-8. In total, 5 studies received a low risk rating, 11 studies received a fair risk rating, and 12 received a high risk rating. Reasons for the ratings were varied, and several studies had multiple reasons for increased risk of bias. Most commonly, studies with fair or high risk scores had issues with blinding (10 studies), high attrition (9 studies), or randomization (7 studies). Blinding issues most commonly revolved around patients and/or providers knowing a patient’s allocation during the study. Randomization issues were varied and included unclear randomization schemes and lack of true randomization (being allocated by study staff). Other less common issues included low sample size (5 studies) and high rates of participant-reported outcomes (5 studies).

## Discussion

### Principal Findings

The findings from this systematic review suggest that available stand-alone mobile phone interventions show some positive changes in behavior and health outcomes in pregnant patients. Although findings were limited and some studies had high risk of bias, these early data suggest such interventions may have some ability to improve behaviors and health outcomes.

With regard to gestational weight gain, obesity, and physical activity, certain interventions did correlate with better health outcomes. In particular, there was less overall gestational weight gain in intervention users over the study period, but all three interventions used a multimodal intervention–either an app or text message in combination with another method of communication, such as social media or email. Regarding smoking cessation, controlled trials did not appear to exhibit an effect of interventions on improved rates of smoking. Regarding influenza vaccination, text message interventions did not improve rates of influenza vaccination. Finally, in the fourth group, which reviewed general prenatal health, interventions were associated with greater knowledge and positive health beliefs, but not with important health outcomes including cesarean delivery or other birth outcomes. Most studies we evaluated exhibited bias, most commonly with unclear blinding, high attrition and poor randomization, though also with low sample sizes, and self-reported outcomes, sometimes from unblinded participants.

Previous research has also explored the multimodal approach in this arena and found it effective. A Dutch study reviewed a 6-month mixed intervention involving Web-based, email, and provider-input components used with 1878 participants who were pregnant or contemplating pregnancy. The usability of the complete program was judged as *positive* or *very positive* by 54.7% of participants and study compliance was 64.86% (1218/1878) among all participants who activated the program. It was also associated with numerous improvements in nutrition and lifestyle behaviors such as improvement in adequate vegetable intake (26.3%, 95% CI 23.0-29.9), fruit intake (38.4%, 95% CI 34.5-42.5), folic acid use (56.3%, 95% CI 48.8-63.6), tobacco abstinence (35.1%, 95% CI 29.1-41.6), and alcohol abstinence (41.9%, 95% CI 35.2-48.9). The strongest effectiveness was for participating couples, which again may point to the multifactorial and social nature of successful interventions [57].

Chronic diseases continue to affect women at high rates during pregnancy and are also associated with increased risk of morbidity later in life, underscoring the need for continued exploration of efficient, wide-reaching interventions for monitoring and behavior modification. A very recent study with a focus on cardiovascular risk in pregnancy and the postpartum period emphasized that women with adverse obstetric outcomes such as preeclampsia, gestational hypertension, and gestational diabetes are at increased risk of developing CVD later in life. Specifically, women with preeclampsia have higher rates of overall CVD (relative risk [RR] 2.15; 95% CI 1.76-2.61), ischemic heart disease (RR 2.06; 95% CI 1.68-2.52), diabetes (RR 2.27; 95% CI 1.55-3.32), and premature cardiovascular death (RR 1.49; 95% CI 1.05-2.14) compared with women with uncomplicated pregnancies. The study authors urge that postpartum women with risk factors should be followed up vigilantly for blood pressure, BMI, waist circumference, lipid profile, fasting glucose, and oral glucose tolerance testing [58]. We propose monitoring and feedback for these patients be included in future mHealth interventions. Further exploration of wearable technologies including smartwatches, fitness bands, and other novel devices may assist with this endeavor.

Further research in this area could take multiple forms. For example, medication adherence for patients with diabetes and hypertension could be explored; a study of adolescents and young adults with sickle cell disease found that of proposed mobile phone app features for improving adherence, daily medication reminders were ranked first most frequently; this sentiment may be shared by pregnant patients in the same age group [59]. Further economic data may also be beneficial. One study of various mHealth interventions with reported economic evaluations found that 74.3% of interventions were cost-effective, economically beneficial, or cost saving [60]. This was briefly noted in one of our reviewed studies [36], but additional data on the topic are necessary. In addition, future work may investigate cross-platform technologies, such as those that are both stand-alone mobile phone platforms and available via the Web.

### Strengths and Limitations

The primary strength of this review is its inclusion of a wide variety of studies that investigated changes in behavior and clinical outcomes, both critical pieces necessary to evaluate novel behavioral technologies. Our study was focused on stand-alone interventions that did not require intensive clinical support and also reviewed data from high-income countries, which may provide more specifically applicable data for patients and physicians.

However, limitations include insufficiency of existing data and lack of granular clinical outcomes data in existing reports. Many included studies also exhibited high levels of bias with an unclear effect on results. In addition, no systematic evaluation of the interventions was performed (for example, using a specified taxonomy), which would allow more formal organization of intervention features themselves. Finally, intervention designs varied widely as did the measured outcomes and time frames of studies. All of these factors precluded the completion of a meaningful meta-analysis.

### Comparison With Existing Literature

A systematic review recently published in April 2018 by Overdijkink et al [61] described a similar review of studies employing text messages and mobile phone apps in pregnancy. Although there was an overlap in included studies, their methodology differed most notably because of the inclusion of telemedicine-based approaches. Most notably, at least five of their studies addressed gestational diabetes telemedicine and remote monitoring systems in which glucometers were coupled with mobile phone communications for nurse or physician feedback. In contrast, we aimed to find studies with minimal clinician input, preferring automated systems, and self-tracking technologies that supported or enhanced behavior changes without added clinician burdens. Furthermore, several of their included studies utilized primarily email and Web-based approaches, whereas we aimed to limit our review to mobile phone app and text-based technologies that could be implemented with use of phones or other primarily mobile technology. Despite these differences, we identified similarly that results are heterogeneous and that additional research is required to evaluate the effects of mHealth interventions on long-standing positive health outcomes.

### Conclusions

In high-income countries, utilization of mobile phone–based health behavior interventions in pregnancy demonstrates some correlation with positive beliefs, behaviors, and health outcomes. More effective interventions are multimodal in terms of features and tend to focus on healthy gestational weight gain. As mHealth interventions become increasingly available, future work must aim to maximize the clinical effectiveness of such interventions. As researchers, we should aim to broaden the scope of effective and sustainable interventions and continue to augment our care with appropriate evidence-based technologies.

## Appendix

Multimedia Appendix 1 Search strategies.

Multimedia Appendix 2 Preferred Reporting Items for Systematic Reviews and Meta-Analysis checklist.

## Abbreviations

CVD: cardiovascular disease
mHealth: mobile health
NIT: National Institutes of Health
NIH QAT: NIH Quality Assessment Tool
PRISMA: Preferred Reporting Items for Systematic Reviews and Meta-Analysis
RCT: randomized controlled trial
RR: relative risk
