# Supplementary material for: Mobile Phone–Based Behavioral Interventions in Pregnancy to Promote Maternal and Fetal Health in High-Income Countries: Systematic Review
Source: JMIR Mhealth Uhealth. 2020 May 28;8(5):e15111. doi: 10.2196/15111 (PMC7290451; doi:10.2196/15111)
Supplement: Multimedia Appendix 1 [file mhealth_v8i5e15111_app1.docx]

Appendix A: Full Search Strategies

We searched PubMed MEDLINE, Embase, Web of Science, Cochrane Database of Controlled Trials, CINAHL, and PsycINFO databases from inception to 8/30/16. We began with the MEDLINE search and translated to the appropriate syntax for each of the other databases.

The search strategies for each of the databases are below.

MEDLINE via PubMed

(((“Pregnancy”[Mesh] OR “pregnancy”[tw] OR “pregnancies”[tw] OR “gestation”[tw] OR “gestational”[tw] OR “parturition”[tw] OR “birth*”[tw] OR “childbirth*”[tw] OR “obstetric labor”[tw] OR “Postpartum period”[Mesh] OR “postpartum”[tw] OR “puerperium”[tw] OR “Perinatal care”[Mesh] OR “perinat*”[tw] OR “postnat*”[tw] OR “pregnant”[tw] OR “trimester”[tw] OR “maternal”[tw] OR “fetus”[Mesh] OR “fetus”[tw] OR “fetuses”[tw] OR “fetal”[tw] OR “Prenatal Care”[Mesh] OR “prenatal”[tw] OR “post-partum”[tw] OR “post partum”[tw] OR “Prenatal education”[Mesh] OR “antenatal” OR “Infant”[Mesh] OR “newborn*”[tw] OR “infant*”[tw] OR “neonate*”[tw] OR “baby”[tw] OR “babies”[tw])) AND (“motor activity”[Mesh] OR “exercise*”[tw] OR “physical activity”[tw] OR “physical activities”[tw] OR “health behavior”[Mesh] OR “health behavior”[tw] OR “health behaviors”[tw] OR “tobacco use cessation”[Mesh] OR “tobacco use cessation”[tw] Or “tobacco cessation”[tw] OR “smoking cessation”[tw] OR “Body mass index”[Mesh] OR “body mass index”[tw] OR “BMI”[tw] OR “Body weight”[Mesh] OR “Diet”[Mesh] OR “diet*”[tw] OR “Food habits”[Mesh] OR “food habits”[tw] OR “food habit”[tw] OR “dietary modification”[tw] OR “dietary modifications”[tw] OR “diet modification”[tw] OR “diet modifications”[tw] OR “dietary habit” OR “dietary habits”[tw] OR “Overweight”[Mesh] OR “overweight”[tw] OR “obesity”[tw] OR “weight loss”[Mesh] OR “weight loss”[tw] OR “weight reduction”[tw] OR “diet records”[Mesh] OR “diet records”[tw] OR “diet record”[tw] OR “Food diary”[tw] OR “food diaries”[tw] OR “dietary record”[tw] OR “dietary records”[tw] OR “calorie counting”[tw] OR “counting calories”[tw] OR “alcohol cessation”[tw] OR “patient compliance”[tw] OR “cooperative behavior”[tw] OR “medication adherence”[tw] OR “no-show patients”[tw] OR “no show patients”[tw] OR “no-show patient”[tw] OR “no show patient”[tw] OR “patient no show”[tw] OR “patient no-show”[tw] OR “hypoglycemia”[Mesh] OR “hypoglycemia”[tw] OR “blood glucose”[Mesh] OR “blood glucose”[tw] OR “blood sugar”[tw] OR “insulin”[Mesh] OR “insulin”[tw] OR “diabetes mellitus”[Mesh] OR “diabetes mellitus”[tw] OR “diabetes”[tw] OR “nutrition”[tw] OR “hyperglycemia”[Mesh] OR “hyperglycemia”[tw] OR “weight gain”[tw])) AND (“mobile health”[tw] OR “mhealth”[tw] OR “ehealth”[tw] OR “m-health”[tw] OR “e-health”[tw] OR “mcare”[tw] OR “Cell Phones”[Mesh] OR “Computers, Handheld”[Mesh] OR “cell phones”[tw] OR “cell phone” OR “cellular phone”[tw] OR “cellular phones”[tw] OR “cellular telephone”[tw] OR “cellular telephones”[tw] OR “mobile phone”[tw] OR “mobile phones”[tw] OR “mobile telephone”[tw] OR “mobile telephones”[tw] OR “iphone”[tw] OR “ipad”[tw] OR “cellphone”[tw] OR “cellphones”[tw] OR “pda”[tw] OR “personal digital assistant”[tw] OR “blackberry”[tw] OR “android”[tw] OR “smartphone”[tw] OR “smartphones”[tw] OR “smart phone”[tw] OR “smart phones”[tw] OR “tablet”[tw] OR “handheld computer”[tw] OR “apps”[tw] OR “mobile application”[tw] OR “mobile applications”[tw] OR “mobile communication”[tw] OR “mobile technology”[tw] OR “mobile games”[tw])

Embase (embase.com)

(‘mobile phone’/exp OR ‘mobile phones’ OR ‘smart phone’ OR ‘smart phones’ OR ‘cell phone’ OR ‘cell phones’ OR ‘cellphone’ OR ‘cellphones’ OR ‘cellular phone’ OR ‘cellular phones’ OR ‘cellular telephone’ OR ‘cellular telephones’ OR ‘mobile telephone’ OR ‘mobile telephones’ OR ‘iphone’ OR ‘ipad’ OR ‘mobile application’ OR ‘mobile applications’ OR ‘mobile app’ OR ‘mobile apps’ OR ‘portable software app’ OR ‘portable software application’ OR ‘portable software apps’ OR ‘personal digital assistant’ OR ‘blackberry’ OR ‘android’ OR ‘tablet’ OR ‘mobile communication’ OR ‘mobile technology’ OR ‘mobile games’ OR ‘microcomputer’ OR ‘microcomputers’) AND (‘pregnancy’/exp OR ‘pregnancies’ OR ‘gestation’ OR ‘child bearing’ OR ‘childbearing’ OR ‘parturition’ OR ‘birth’ OR ‘childbirth’/exp OR ‘labor’/exp OR ‘labour’ OR ‘puerperium’/exp OR ‘postpartum’ OR ‘post partum’ OR ‘puerperal’ OR ‘trimester’ OR ‘pregnant’ OR ‘maternal’ OR ‘fetus’ OR ‘fetuses’ OR ‘fetal’ OR ‘prenatal care’/exp OR ‘ante natal care’ OR ‘antenatal care’ OR ‘antenatal control’ OR ‘infant’/exp OR ‘baby’ OR ‘babies’ OR ‘infants’ OR ‘neonate’ OR ‘childbirth education’ OR ‘prenatal education’) AND (‘exercise’/exp OR ‘physical activity’/exp OR ‘health behavior’/exp OR ‘smoking cessation’ OR ‘quit smoking’ OR ‘stop smoking’ OR ‘tobacco use cessation’ OR ‘body mass’ OR ‘body mass index’ OR ‘BMI’ OR ‘body weight’/exp OR ‘diet’/exp OR ‘feeding behavior’/exp OR ‘food habit’ OR ‘nutrition pattern’ OR ‘eating behavior’ OR ‘obesity’/exp OR ‘overweight’ OR ‘weight reduction’/exp OR ‘weight loss’ OR ‘diet restriction’/exp OR ‘calorie restriction’ OR ‘alcohol abstinence’ OR ‘alcohol cessation’ OR ‘medication compliance’ OR ‘medication adherence’ OR ‘patient attendance’ OR ‘no-show patient’ OR ‘non-attending patient’ OR ‘hypoglycemia’/exp OR ‘hyperglycemia’ OR ‘glucose blood level’ OR ‘blood glucose’ OR ‘blood sugar’ OR ‘insulin’ OR ‘diabetes mellitus’/exp)

Web of Science

TS=exercis* OR

TS=“health behavior” OR

TS=“body mass index” OR

TS=“bmi” OR

TS=“body weight” OR

TS=“diet” OR

TS=“food habits” OR

TS=“obesity” OR

TS=“body weight” OR

TS=“diet records” OR

TS=hypoglycemia OR

TS=hyperglycemia OR

TS=“blood glucose” OR

TS=insulin OR

TS=diabetes OR

TS=“physical activity” OR

TS=“smoking cessation” OR

TS=“tobacco use cessation” OR

TS=diet* OR

TS=“weight loss” OR

TS=overweight OR

TS=“calorie counting” OR

TS=“counting calories” OR

TS=“alcohol cessation” OR

TS= “patient compliance” OR

TS=“medication adherence” OR

TS=“patient no-show” OR

TS=“no-show patient”

AND

TS=“cellular phone” OR

TS=“cell phone” OR

TS=“cell phones” OR

TS=smartphone* OR

TS=“tablet computer” OR

TS=“mobile phone” OR

TS=“mobile phones” OR

TS=iphone* OR

TS=ipad* OR

TS=blackberry OR

TS=android OR

TS=“personal digital assistant” OR

TS=“mobile application” OR

TS=“mobile applications” OR

TS=“mobile app” OR

TS=“mobile apps” OR

TS=“mobile game” OR

TS=“mobile games”

AND

TS=preganc* OR

TS=“postnatal period” OR

TS=“obstetric care” OR

TS=fetus* OR

TS=infant* OR

TS=“childbirth education” OR

TS=gestation OR

TS=parturition OR

TS=birth* OR

TS=childbirth* OR

TS=“obstetric labor” OR

TS=postpartum OR

TS=perinat* OR

TS=postnat* OR

TS=puerperium OR

TS=pregnant OR

TS=trimester* OR

TS=newborn* OR

TS=baby OR

TS=babies OR

TS=neonat*

CENTRAL (Wiley)

ID Search

#1 MeSH descriptor: [Pregnancy] explode all trees

#2 MeSH descriptor: [Postpartum Period] explode all trees

#3 MeSH descriptor: [Perinatal Care] explode all trees

#4 MeSH descriptor: [Fetus] explode all trees

#5 MeSH descriptor: [Prenatal Care] explode all trees

#6 MeSH descriptor: [Prenatal Education] explode all trees

#7 MeSH descriptor: [Infant] explode all trees

#8 "pregnancy" or "pregnancies" or "gestation" or "gestational" or "parturition" or "birth*" or "childbirth*" or "obstetric labor" or "postpartum" or "puerperium" or "perinat*" or "postnat*" or "pregnant" or "trimester" or "maternal" or "fetus" or "fetuses" or "fetal" or "prenatal" or "post-partum" or "post partum" or "antenatal" or "newborn*" or "infant*" or "neonate*" or "baby" or "babies"

#9 MeSH descriptor: [Motor Activity] explode all trees

#10 MeSH descriptor: [Health Behavior] explode all trees

#11 MeSH descriptor: [Tobacco Use Cessation] explode all trees

#12 MeSH descriptor: [Body Mass Index] explode all trees

#13 MeSH descriptor: [Body Weight] explode all trees

#14 MeSH descriptor: [Diet] explode all trees

#15 MeSH descriptor: [Food Habits] explode all trees

#16 MeSH descriptor: [Overweight] explode all trees

#17 MeSH descriptor: [Weight Loss] explode all trees

#18 MeSH descriptor: [Diet Records] explode all trees

#19 MeSH descriptor: [Hypoglycemia] explode all trees

#20 MeSH descriptor: [Blood Glucose] explode all trees

#21 MeSH descriptor: [Insulin] explode all trees

#22 MeSH descriptor: [Diabetes Mellitus] explode all trees

#23 MeSH descriptor: [Hyperglycemia] explode all trees

#24 "exercise*" or "physical activity" or "physical activities" or "health behavior" or "health behaviors" or "tobacco use cessation" or "tobacco cessation" or "smoking cessation" or "body mass index" or "BMI" or "diet*" or "food habits" or "food habit" or "dietary modification" or "dietary modifications" or "diet modification" or "diet modifications" or "dietary habit" or "dietary habits" or "overweight" or "obesity" or "weight loss" or "weight reduction" or "diet records" or "diet record" or "Food diary" or "food diaries" or "dietary record" or "dietary records" or "calorie counting" or "counting calories" or "alcohol cessation" or "patient compliance" or "cooperative behavior" or "medication adherence" or "no-show patients" or "no show patients" or "no-show patient" or "no show patient" or "patient no show" or "patient no-show" or "hypoglycemia" or "blood glucose" or "blood sugar" or "insulin" or "diabetes mellitus" or "diabetes" or "nutrition" or "hyperglycemia" or "weight gain"

#25 MeSH descriptor: [Cell Phones] explode all trees

#26 MeSH descriptor: [Computers, Handheld] explode all trees

#27 MeSH descriptor: [Text Messaging] explode all trees

#28 "mobile health" or "mhealth" or "ehealth" or "m-health" or "e-health" or "mcare" or "cell phones" or "cell phone" or "cellular phone" or "cellular phones" or "cellular telephone" or "cellular telephones" or "mobile phone" or "mobile phones" or "mobile telephone" or "mobile telephones" or "iphone" or "ipad" or "cellphone" or "cellphones" or "pda" or "personal digital assistant" or "blackberry" or "android" or "smartphone" or "smartphones" or "smart phone" or "smart phones" or "tablet" or "handheld computer" or "text message" or "text messaging" or "texting" or "text" or "texts" or "SMS" or "short message service" or "apps" or "mobile application" or "mobile applications" or "mobile communication" or "mobile technology" or "mobile games"

#29 {or #1-#8}

#30 {or #9-#24}

#31 {or #25-#28}

#32 #29 and #30 and #31

CINAHL (EBSCO)

((MH “Pregnancy+”) OR (MH “Postnatal Period+”) OR (MH “Obstetric Care+”) OR (MH “fetus+”) OR (MH “Infant+”) OR (MH “Childbirth education”) OR TI pregnancy OR AB pregnancy OR TI pregnancies OR AB pregnancies OR TI gestation OR AB gestation* OR TI gestation* OR TI parturition OR AB parturition OR TI birth* OR AB birth* OR TI childbirth* OR AB childbirth* OR TI ”obstetric labor” OR AB “obstetric labor” OR TI postpartum OR AB postpartum OR TI puerperium OR AB puerperium OR TI perinat* OR AB perinat* OR TI postnat* OR AB postnat* OR TI pregnant OR AB pregnant OR TI trimester OR AB trimester OR TI maternal OR AB maternal OR TI fetus* OR AB fetus* OR TI antenatal OR AB antenatal OR TI “post partum” OR AB “post partum” OR TI infant* OR AB infant* OR TI newborn* OR AB newborn* OR TI neonat* OR AB neonat* OR TI baby OR AB baby OR TI babies or AB babies) AND

((MH “Exercise+”) OR (MH “health behavior+”) OR (MH “Body mass index”) OR (MH “body weight”) OR (MH “Diet+”) OR (MH “food habits+”) OR (MH “obesity+”) OR (MH “body weight changes+”) OR (MH “diet records”) OR (MH “hypoglycemia+”) OR (MH “hyperglycemia”) OR (MH “blood glucose”) OR (MH “insulin+”) OR (MH “diabetes mellitus+”) OR TI excercis* OR AB excercis* OR TI “physical activity” OR AB “physical activity” OR TI “health behavior” OR AB “health behavior” OR TI “tobacco use cessation” OR AB “tobacco use cessation” OR TI “smoking cessation” OR AB “smoking cessation” OR TI “body mass index” OR AB “body mass index” OR TI BMI or AB BMI OR TI “body weight” OR AB “body weight” OR TI diet* OR AB diet* OR TI “food habits” OR AB “food habits” OR TI “dietary modifications” OR AB “dietary modifications” OR TI “weight loss” OR AB “weight loss” OR TI overweight or AB overweight OR TI “weight reduction” OR AB “weight reduction” OR TI “diet records” OR AB “diet records” OR TI “calorie counting” OR AB “calorie counting” OR TI “counting calories” OR AB “counting calories” OR TI “alcohol cessation” OR AB “alcohol cessation” OR TI “patient compliance” OR AB “patient compliance” OR TI “medication adherence” OR AB “medication adherence” OR TI “patient no show” OR AB “patient no show” OR TI “no-show patient” OR AB “no-show patient” OR TI hyperglycemia OR AB hyperglycemia OR TI hypoglycemia OR AB hypoglycemia OR TI “blood glucose” OR AB “blood glucose” OR TI insulin OR AB insulin OR TI diabetes OR AB diabetes) AND ((MH “cellular phone+) OR (MH “computers, hand-held+”) OR TI “cell phone” OR AB “cell phone” OR TI “cell phones” OR AB “cell phones” OR TI “cellular phone” OR AB “cellular phone” OR TI “cellular phones” OR AB “cellular phones” OR TI “mobile phone” OR AB “mobile phone” OR TI “mobile phones” OR AB “mobile phones” OR TI iphone* OR AB iphone* OR TI ipad* OR AB ipad* OR TI cellphone* OR AB cellphone* OR TI “personal digital assistant” OR AB “personal digital assistant” OR TI smartphone* OR AB smartphone* OR TI “text messag*” OR AB “text messag*” OR TI texting or AB texting OR TI “mobile application*” OR AB “mobile application*” OR TI “mobile game*” OR AB “mobile game*” OR TI blackberry OR AB blackberry OR TI android or AB android)

PsycINFO (EBSCO)

((DE “Pregnancy+”) OR (DE “Postnatal Period+”) OR (DE “Obstetric Care+”) OR (DE “fetus+”) OR (DE “Infant+”) OR (DE “Childbirth education”) OR TI pregnancy OR AB pregnancy OR TI pregnancies OR AB pregnancies OR TI gestation OR AB gestation* OR TI gestation* OR TI parturition OR AB parturition OR TI birth* OR AB birth* OR TI childbirth* OR AB childbirth* OR TI ”obstetric labor” OR AB “obstetric labor” OR TI postpartum OR AB postpartum OR TI puerperium OR AB puerperium OR TI perinat* OR AB perinat* OR TI postnat* OR AB postnat* OR TI pregnant OR AB pregnant OR TI trimester OR AB trimester OR TI maternal OR AB maternal OR TI fetus* OR AB fetus* OR TI antenatal OR AB antenatal OR TI “post partum” OR AB “post partum” OR TI infant* OR AB infant* OR TI newborn* OR AB newborn* OR TI neonat* OR AB neonat* OR TI baby OR AB baby OR TI babies or AB babies) AND

((DE “Exercise+”) OR (DE “health behavior+”) OR (DE “Body mass index”) OR (DE “body weight”) OR (DE “Diet+”) OR (DE “food habits+”) OR (DE “obesity+”) OR (DE “body weight changes+”) OR (DE “diet records”) OR (DE “hypoglycemia+”) OR (DE “hyperglycemia”) OR (DE “blood glucose”) OR (DE “insulin+”) OR (DE “diabetes mellitus+”) OR TI excercis* OR AB excercis* OR TI “physical activity” OR AB “physical activity” OR TI “health behavior” OR AB “health behavior” OR TI “tobacco use cessation” OR AB “tobacco use cessation” OR TI “smoking cessation” OR AB “smoking cessation” OR TI “body mass index” OR AB “body mass index” OR TI BMI or AB BMI OR TI “body weight” OR AB “body weight” OR TI diet* OR AB diet* OR TI “food habits” OR AB “food habits” OR TI “dietary modifications” OR AB “dietary modifications” OR TI “weight loss” OR AB “weight loss” OR TI overweight or AB overweight OR TI “weight reduction” OR AB “weight reduction” OR TI “diet records” OR AB “diet records” OR TI “calorie counting” OR AB “calorie counting” OR TI “counting calories” OR AB “counting calories” OR TI “alcohol cessation” OR AB “alcohol cessation” OR TI “patient compliance” OR AB “patient compliance” OR TI “medication adherence” OR AB “medication adherence” OR TI “patient no show” OR AB “patient no show” OR TI “no-show patient” OR AB “no-show patient” OR TI hyperglycemia OR AB hyperglycemia OR TI hypoglycemia OR AB hypoglycemia OR TI “blood glucose” OR AB “blood glucose” OR TI insulin OR AB insulin OR TI diabetes OR AB diabetes) AND ((DE “cellular phone+) OR (DE “computers, hand-held+”) OR TI “cell phone” OR AB “cell phone” OR TI “cell phones” OR AB “cell phones” OR TI “cellular phone” OR AB “cellular phone” OR TI “cellular phones” OR AB “cellular phones” OR TI “mobile phone” OR AB “mobile phone” OR TI “mobile phones” OR AB “mobile phones” OR TI iphone* OR AB iphone* OR TI ipad* OR AB ipad* OR TI cellphone* OR AB cellphone* OR TI “personal digital assistant” OR AB “personal digital assistant” OR TI smartphone* OR AB smartphone* OR TI “text messag*” OR AB “text messag*” OR TI texting or AB texting OR TI “mobile application*” OR AB “mobile application*” OR TI “mobile game*” OR AB “mobile game*” OR TI blackberry OR AB blackberry OR TI android or AB android)
